# The Limitations of a Voluntary Register

**Published:** 1920-09-11

**Authors:** 


### THE MATRONS' AND SISTERS' DEPARTMENT.

### THE LIMITATIONS OF A VOLUNTARY REGISTER.

In the old days when State registration was much talked about but little understood, many of the nurses who agitated for it did so under the impression that it would be compulsory. Even now it seems to be a shock to some that the State, having placed this privilege within their grasp, can exercise no pressure upon nurses to register their names. Truth to tell, there is ground for some misapprehension. The only Register of which nurses have practical cognisance is that of the Central Mdwives Board, and there is nothing voluntary about that. Midwives may not practise at all unless their names are inscribed on it. It is certainly very puzzling to find that the State Registration Board cannot also insist upon all nurses coming within the fold.

In one respect there will be even less compulsion for nurses than for doctors to be registered. Doctors are practically under compulsion in this respect, because unless their names are on the register the State will not give them the benefit of the law in collecting their debts. A man cannot be sued for medical attendance by a quack.

It has, however, never been deemed possible to set up a compulsory register for nurses, because Parliament will not consent to make it a penal offence for women whose names were not on the register to act as nurses. Nursing is too universal an office to be restricted by hard-and-fast rules. The woman who comes short of the qualifications required by the Board may be indispensable to the health of some one. To restrict her from aiding her neighbour in sickness would be an injustice to her and a cruelty to her patient. However incapable, she might be better than no one at a time of extremity, and it would be a drastic thing to prohibit her, even "habitually and for gain," from nursing altogether.

The result of making the register voluntary is this. It sets up a kingdom within a kingdom. Within the enormous company of practising nurses of every degree it distinguishes an inner ring of women chosen out by reason of their superior attainments and their high character. It confers upon them a certain status, the value of which will depend entirely upon the number and quality of those who enter it. For this inner ring of registered nurses it establishes exact regulations and prohibitions. It exercises a certain discipline over them, and in the last resort can eject them from the register. It guarantees to the public that a tolerable standard of professional knowledge has been acquired by all whom it admits. But alas, it must leave all the other nurses who neglect to get their names inscribed on the register severely alone. Here is the grave defect of the voluntary register. It keeps watch over the elect. It lets the runagates continue in scarceness—of grace.

The complete liberty allowed under State registration to the nurses who remain outside its jurisdiction is a very serious impediment to organisation. To a large extent it neutralises the public value of the register. So far as the complete regulation of the nursing profession goes this voluntary principle may be said to defeat its aims altogether. For who are the persons who will be benefited by having a State register? Are they not the rich, or at least the well-to-do, able to pay a good fee? But the people who most need protection are the middle classes. And it is among these that the nurses not on the register will naturally find their *clientèle* and flourish uninspected, unlicensed, uncontrolled. It is doubtful whether many of the rich will not also succumb to the voice of the charmer, demonstrating glibly that she is just as good, nay, far better, than the registered nurses.

It is well that nurses should be on their guard from the beginning against the dangers which will accrue to them from the unregistered. There is but one way of making the voluntary principle work. It is for all entitled to do so to apply for registration. If the voluntary State register be cordially accepted by every single nurse of repute, then the outsiders can do little harm. They will be self-exposed. Nothing but a wave of sincere enthusiasm for the new privilege accorded it can carry the nursing profession into the haven where it would be. Every well-trained nurse who stands aloof will supply an argument for the acceptance of the incompetent, by whose side she places herself in rejecting registration.

Whether eventually some system can be devised for licensing and inspecting women not on the register who "habitually and for gain" practice nursing is a matter which sooner or later will have to be faced.

